# Tissue Element Levels and Heavy Metal Burdens in Bottlenose Dolphins That Stranded in the Mississippi Sound Surrounding the 2019 Unusual Mortality Event

**DOI:** 10.3390/toxics13060511

**Published:** 2025-06-18

**Authors:** Nelmarie Landrau-Giovannetti, Ryanne Murray, Stephen Reichley, Debra Moore, Theresa Madrigal, Ashli Brown, Ashley Meredith, Christina Childers, Darrell Sparks, Moby Solangi, Anna Linhoss, Beth Peterman, Mark Lawrence, Barbara L. F. Kaplan

**Affiliations:** 1 Global Center for Aquatic Health and Food Security, Mississippi State University, Mississippi State, MS 39762, USA; nelmarie.landrau@gmail.com (N.L.-G.); rm2483@msstate.edu (R.M.); stephen.reichley@msstate.edu (S.R.); debra.moore@msstate.edu (D.M.); aep9@msstate.edu (B.P.); lawrence@cvm.msstate.edu (M.L.); 2 Department of Pathobiology and Population Medicine, College of Veterinary Medicine, Mississippi State University, Mississippi State, MS 39762, USA; 3 Institute for Marine Mammal Studies, Gulfport, MS 39503, USA; tmadrigal@imms.org (T.M.); moby@imms.org (M.S.); 4 Mississippi State Chemical Laboratory, Mississippi State University, Mississippi State, MS 39762, USA; ab506@msstate.edu (A.B.); ameredith@mslc.msstate.edu (A.M.); cchilders@mscl.msstate.edu (C.C.); darrell.sparks@msstate.edu (D.S.); 5 Department of Biosystems Engineering, Samuel Ginn College of Engineering, Auburn University, Auburn, AL 36849, USA; alinhoss@auburn.edu; 6 Department of Comparative Biomedical Sciences, College of Veterinary Medicine, Mississippi State University, Mississippi State, MS 39762, USA; 7 Center for Environmental Health Sciences, College of Veterinary Medicine, Mississippi State University, Mississippi State, MS 39762, USA

**Keywords:** dolphins, Mississippi Sound, metals, unusual mortality event, Bonnet Carré Spillway

## Abstract

In 2019, an unusual mortality event (UME) affected bottlenose dolphins (*Tursiops truncatus*) in the Mississippi Sound (MSS) following an extended dual opening of the Bonnet Carré Spillway (BCS), which prevents flooding in New Orleans. This resulted in low salinity, skin lesions, and electrolyte imbalances in dolphins. Additionally, the influx likely altered the MSS’s environmental chemical composition, including levels of heavy metals and metalloids; thus, we quantified heavy metals, metalloids, and essential elements in the tissues of dolphins that stranded in the MSS before and after the 2019 UME. We hypothesized that levels of heavy metals and metalloids (such as mercury (Hg), arsenic (As), lead (Pb), and cadmium (Cd)) would not show significant changes post-UME. Indeed, we found no major changes associated with the UME in most metals; sodium (Na) and magnesium (Mg) levels were lower in several tissues after 2019, which correlated with the average yearly salinity measured from the MSS. Toxic metals and metalloids were detectable with some changes over time; however, the selenium (Se):Hg molar ratio increased in some tissues post-2019. Additionally, we confirmed that Hg can bioaccumulate, with positive correlations between Hg levels and dolphin size as assessed by straight length. Overall, our findings indicate that freshwater incursions into the MSS can alter dolphin exposure to essential and toxic elements.

## 1. Introduction

There are several metals and metalloids that are essential elements, which include sodium (Na), potassium (K), magnesium (Mg), calcium (Ca), manganese (Mn), iron (Fe), cobalt (Co), copper (Cu), zinc (Zn), and molybdenum (Mo) [1]. A few of these essential and other minor elements might also produce disease at low levels, but exhibit toxic effects at elevated levels, including Co, Cu, Fe, Mg, Mn, Zn, nickel (Ni), chromium (Cr), and selenium (Se) [2], although whether Cr should even be considered a minor essential element has been debated [3]. Other metals commonly found in the environment through natural or anthropogenic sources exhibit toxic effects even at low levels, such as the metals/metalloids mercury (Hg), lead (Pb), cadmium (Cd), and arsenic (As). Heavy metals are defined by the fact that they have relatively high density, atomic weights, or atomic numbers; heavy metals typically refer to As, Cd, chromium (Cr), Pb, Hg, and thallium (Tl) [4].

Metals are released into the marine environment as byproducts of several industrial operations, contributing varying amounts of different metals [5,6]. The list of metals considered toxic and of concern has been largely—though not entirely—restricted to the ten that seem to be the most lethal to marine life. They are Hg, Cd, silver (Ag), Ni, Se, Pb, Cu, Cr, As, and Zn, listed in decreasing order of toxicity [6,7]. Heavy metals are also contaminants of concern for marine habitats because some exhibit bioaccumulation [8].

One of the most populated marine habitats for bottlenose dolphins (*Tursiops truncatus*) is in the Mississippi Sound (MSS) [9,10,11]. Exposure of dolphins to various metals, metalloids, and other elements varies with temperature, season, weather, and water levels. In the MSS, salinity and other water parameters can also change drastically with freshwater incursions into the sound from the Mississippi (MS) and other feeder rivers. In fact, as part of the flood control system for the city of New Orleans, the Bonnet Carré Spillway (BCS), located about 12 miles west of the city, can be opened during periods of heavy rain to divert water into Lake Pontchartrain and the MSS [12]. Usually, the BCS only requires opening no more than once per year, but in 2019, it was opened twice (for a 43-day period followed by a 79-day period), allowing over ten trillion gallons of freshwater to be diverted into Lake Pontchartrain and the MSS [12,13]. This diversion caused a bottlenose dolphin unusual mortality event (UME) in the MSS as a result of exposure to low-salinity waters [14].

The objectives of this study were to determine the levels of metals (essential and non-essential) and metalloids in tissues from dolphins that stranded along the MSS from 2010 to 2021. This was an opportunistic, retrospective quantitative analysis of blubber, kidney, liver, and muscle from stranded dolphins that allowed us to assess whether there were any changes in metals or metalloids surrounding the 2019 UME. We partitioned the data over four time periods: tissues obtained from strandings that occurred before 2019 (b. 2019), then by year thereafter for 2019, 2020, and 2021. Thus, our specific goals were to (1) quantify the time-dependent changes in essential metals and metalloids, (2) quantify the time-dependent changes in toxic and heavy metals and metalloids, and (3) determine if a correlation exists between concentrations of Hg and dolphin straight length.

## 2. Materials and Methods

### 2.1. Dolphin Selection Criteria

The tissue samples examined are the same as those used to quantify levels of other environmental chemicals, such as PCBs and PAHs, which have been published [15]. Bottlenose dolphin necropsies and tissues collected for toxicology analysis in this study were conducted under the Marine Mammal Stranding Agreement between the Southeast Region National Marine Fisheries Service of the National Oceanic and Atmospheric Administration (NOAA) and the Institute for Marine Mammal Studies (IMMS). In addition, IMMS has a cooperative agreement with the Mississippi Department of Marine Resources (MDMR) acting as an agent under its Section 6 cooperative agreement with NOAA and under Section 109 (h) (1) of the Marine Mammal Protection Act to respond to distressed and stranded marine mammals in the state of Mississippi. Tissue samples were collected from dolphin carcasses classified as code 2 (fresh dead carcass with no smell or bloating) or code 3 (carcass with moderate decomposition of varying levels: early, mid, or late) [16] stranded along the MSS from 2010 to 2021. It is important to note that these samples were obtained in an opportunistic, not systematic, manner, and therefore, they do not represent all dolphins that were stranded during this time period.

### 2.2. Sample Preparation and Extraction Method

Sample preparation from blubber, kidney, liver, and muscle was previously described [15]. Individual samples were obtained from 42, 43, 24, and 37 dolphins that stranded from 2010 to 2018, 2019, 2020, and 2021, respectively, for a total of 146 (with the exception that one kidney sample was missing from 2021, making the total 145 in 2021). Each individual dolphin had matched samples of blubber, kidney, liver, and muscle. Prior to the extraction and analysis of the tissues, samples were partially thawed and processed with various techniques. In order to homogenize the tissue samples, a range of specialized stainless steel tools were used to provide uniformity and maximize the efficiency of subsequent testing. The choice of tool and extent of preparation depended on the nature of the sample and the sample amount available. For limited sample sizes, disposable scalpels (Fisher Scientific) and/or sterile single-use scissors (Fisher Scientific) were used to finely mince the tissues. Tissue samples with more available mass were processed with traditional cutting knives and a commercial meat grinder (Hobart, Troy, OH), composed of food-grade stainless steel (grade 304).

### 2.3. Reagents and Materials

All reagents and gases used in this study were of ultra-high-purity, trace-metal grade to minimize contamination risk and were verified to be free of target analytes prior to use. Ultrapure water (resistivity of 18 MΩ cm) was generated with a Milli-Q IQ 7000 Ultrapure Water Purification System (MilliporeSigma, Burlington, MA, USA). The internal standard mix and environmental calibration standard (ECS) were obtained from Agilent Technologies (Santa Clara, CA, USA). The ECS contained 1000 ppm each of Fe, K, CA, Na, Mg, and 10 ppm each of Ag, Al, As, Ba, Be, Cd, Co, Cr, Cu, Mn, Mo, Ni, Pb, Sb, Se, Tl, V, Zn, Th, U prepared in 10% nitric acid. The internal standard mix contained 100 ppm of 6-Li, Sc, Ge, Rh, In, Tb, Lu, Bi, also prepared in 10% nitric acid. Hydrogen peroxide solutions (30%) for trace analysis were purchased from Millipore Sigma (Burlington, MA, USA) in small, 500 mL volumes to prevent degradation before use. Trace metal grade nitric and hydrochloric acids were provided by Fisher Scientific (Waltham, VA, USA), as well as Triton X-100. Helium and Argon gases used for Inductively Coupled Plasma–Mass Spectrometry (ICP-MS) analysis were purchased from nexAir (Memphis, TN, USA), and certified with a minimum purity of 99.999%.

### 2.4. Extraction Method

The extraction of metals from tissue samples was performed using accelerated acid microwave digestion to ensure efficient breakdown of the tissue sample and effective analyte release from each matrix. A MARS 6 (CEM, Charlotte, NC, USA) microwave digestion unit was employed for this procedure. Individual microwave vessels containing 0.250 g (±0.001) of homogenized tissue were fortified with 7.5 mL of trace-grade nitric acid (Fisher Scientific). Additionally, 2.5 mL of 30% hydrogen peroxide solution (Millipore/Sigma) was added to each vessel to aid in the oxidation of the organic matter and accelerate the digestion process. Quality control samples consisting of bovine tissues, provided by the MSU Animal and Dairy Sciences Department, were analyzed with every batch of 37 samples. Bovine tissues were selected as quality control materials instead of marine certified reference materials (CRMs) due to their closer physiological and anatomical similarities to dolphins. Both dolphins and bovines are placental mammals that share a common terrestrial lineage, which contributes to similarities in tissue composition and biological responses to stressors, including environmental contaminants [17,18]. These mammalian parallels provide a more appropriate matrix for evaluating digestion efficiency, recovery, and instrument performance than non-mammalian aquatic CRMs such as fish muscle or liver, particularly in the absence of species-specific marine mammal CRMs. The MARS 6 microwave carousel allows for the simultaneous digestion of 40 vessels; therefore, the carousel included 36 tissue samples, a lab reagent blank, a bovine matrix spike, and duplicates of the bovine matrix unfortified. To evaluate extraction efficiency, fortified bovine tissues were prepared by adding a known quantity of a multi-element analytical standard (Agilent Technologies) prior to digestion. Recovery was calculated by comparing the measured concentration of each target element in the fortified tissue to the expected concentration based on the fortification level. Acceptable recovery was defined as 80–120%. If quality control spikes did not come within tolerance, or the reagent blank displayed contamination above the quantitation level for the corresponding element, the 36 samples within the batch were re-processed. Once the batches were prepared and placed in the carousel, the vessels underwent a heating program in which the temperature gradually increased to 200 °C over a period of 15 min and was subsequently maintained at 200 °C for an additional 20 min to ensure complete digestion. Following digestion, the samples were cooled, and the resulting solutions were brought to a final volume of 50 mL using ultrapure water. Approximately 10 mL of the digestion extract was then filtered through a Millex™ 33 mm 0.45 µm PVDF filter (Fisher Scientific) to remove any particulate matter. Lastly, the filtrate was collected into a polypropylene autosampler vial (Agilent Technologies) for subsequent analysis. Metal concentrations in the extracts were quantitated using an Agilent 7900 Inductively Coupled Plasma-Mass Spectrometer (ICP-MS), providing high sensitivity and precision for trace metal quantification.

### 2.5. ICP-MS Method Validation

Method validation included the determination of limits of detection (LOD) and limits of quantitation (LOQ), calculated from seven replicate blank measurements. LODs were defined as the minimum concentration with 99% confidence that the measured value was above zero, and LOQs were determined by applying the extraction dilution factor to the LOD determined. Intra-assay precision (repeatability) and inter-assay precision (reproducibility) were assessed using seven replicate measurements at three fortification levels (low, medium, and high) for each tissue type. Relative standard deviations (%RSD) across all analytes ranged from 0.3% to 8.0%, with mean recoveries between 88% and 109%. The LODs and LOQs were 0.00039/0.039 µg/g wet weight (ww) for Al, As, Ba, Cd, Cr, Cu, Fe, Pb, Mn, Ni, Se, Ag, and Zn; 0.0006/0.0006 µg/g ww for Hg; and 0.390/3.90 µg/g ww for Ca, Mg, P, K, and Na. All analytes demonstrated strong linearity, with correlation coefficients (r^2^) ranging from 0.998 to 1.000. The quantitation of metal concentrations was calculated with the use of a matrix-matched serial diluted calibration curve made from the ECS (Agilent Technologies, Santa Clara, CA, USA) in ultrapure water with 2% trace-grade nitric acid. The calibration curve was prepared weekly and ranged from 0.391 to 100 ng/mL for trace metals and 39.1 to 10,000 ng/mL for minerals with 9 calibration points included. The calibration curve coefficient for all elements was required to be at or above an R^2^ of 0.995. The ICP-MS was optimized, prior to analysis, with a tuning solution containing Li, Mg, Y, Ce, Tl, and Co (Agilent Technologies, Santa Clara, CA, USA). A reference material (Waters^TM^ ERA^TM^, (S308-697; Golden, CO, USA) was also analyzed prior to the samples to trend instrument and calibration performance by use of control charts. An Agilent 7900 ICP-MS coupled to an SPS 4 Autosampler (Agilent Technologies) with standard nickel cones was used to complete the analysis of metals in the tissues. The SPS 4 autosampler was coupled to a sample introduction system including a peristaltic pump for precise sample introduction at 0.30 rps. The peri pump was equipped with a sample line (Agilent Technologies, Santa Clara, CA, USA), a waste line (Agilent Technologies, Santa Clara, CA, USA), and an internal standard line (Agilent Technologies, Santa Clara, CA, USA). The internal standard (Agilent Technologies, Santa Clara, CA, USA) was used to correct for variations in sample matrices and instrument performance and included 6-Li, Sc, Ge, Rh, In, Tb, Lu, Bi. The instrument method included a rinse program that incorporated three rinses of the sample probe between each sample to minimize contamination. A rinse program included a series of three rinses, consisting of 2% nitric acid, 0.5% hydrochloric acid, and 0.01% Triton, of the sample probe between samples to limit contamination between samples. The peri pump was equipped with a 1.02 mm id sample line (Agilent Technologies), a 1.52 mm id waste line (Agilent Technologies, Santa Clara, CA, USA), and a 0.25 mm id internal standard line (Agilent Technologies). The internal standard was used to correct variations in sample matrices and instrument performance. The ICP-MS was operated in general-purpose plasma mode with the following operating parameters: radio frequency power of 1550 W, plasma gas flow of 10 L/min, auxiliary gas flow of 1.05 L/min, and nebulizer gas flow of 0.99 L/min. The dwell time for each mass was optimized to ensure adequate signal intensity and sensitivity. All measurements were performed in triplicate from each injection. Helium gas was introduced into the collision cell to reduce polyatomic interferences caused by the complex organic matrices. Instrument blanks and calibration verifications were analyzed every 20 sample injections to monitor instrument stability and ensure data accuracy. The data collected from the ICP-MS were processed and reported using MassHunter software 5.2 (Version D.01.02) (Agilent Technologies, Santa Clara, CA, USA).

### 2.6. Calculations and Statistics

The levels in this study are expressed as μg g^−1^ wet weight (ww), and the ww values were used in all statistical analyses. If there were duplicate tissues from an individual, the average μg g^−1^ was calculated. Se and Hg molar ratios were calculated according to [19]. Briefly, the tissue concentrations of Se and Hg were divided by their molecular weights (78.96 for Se and 200.59 for Hg) from which molar ratios were calculated for any tissue in which both Se and Hg were detected. Normality tests were conducted using the Shapiro–Wilk test. At least one time group within each metal/metalloid and tissue was deemed non-normal except K in kidney (although a comparison of the one-way ANOVA followed by Tukey’s test versus Kruskal–Wallis followed by Dunn’s test revealed the same pattern in the differences detected). Thus, time period differences with each metal/metalloid and tissue were reported using Kruskal–Wallis non-parametric analysis with a *p* < 0.05 followed by Dunn’s test to detect differences. Sex differences were detected using a Mann–Whitney test within each chemical and tissue. Correlations were calculated using Pearson’s correlation coefficients. All statistical analyses were performed using GraphPad Prism software v9.5.1 (GraphPad Software, San Diego, CA, USA).

## 3. Results

In total, 22 metals or elements were quantified in blubber, kidney, liver, and muscle from dolphins stranded along the coast of the MSS from 2010 to 2021. Results from beryllium (Be), antimony (Sb), and Tl will not be shown since there was only one sample that was positive for any of these elements (Sb in one kidney from 2021; Supplemental Table S1). We detected statistically significant sex differences in Cr (male, 0.28 ± 26 µg/g versus female, 0.57 ± 0.55 µg/g) and Mn (male, 0.57 ± 1.5 µg/g versus female, 0.41 ± 0.35 µg/g) in blubber, and Mg (male, 203 ± 51 µg/g versus female, 228 ± 91 µg/g) in muscle (Supplemental Table S1).

### 3.1. Elemental Analysis

First, we assessed various elements in tissues from stranded dolphins over time. Since these data are extensive, we have grouped the data according to essential elements and non-essential toxic and/or heavy metals, with Hg and Se presented separately. Many of the essential elements’ results are summarized in Table 1 with individual graphs provided (Supplemental Figures S1–8).

There were statistically significant time-dependent (T-D) effects of essential metals and metalloids detected in the various tissues (Table 1). For instance, Ca and Cu were lowest in liver in 2019 and K was lower in kidney in 2019 as compared to b. 2019. For the rest of the elements above, we do not consider the T-D changes related to the dual BCS opening in 2019 because the T-D effects did not involve the 2019 timeframe.

**Figure 1:**
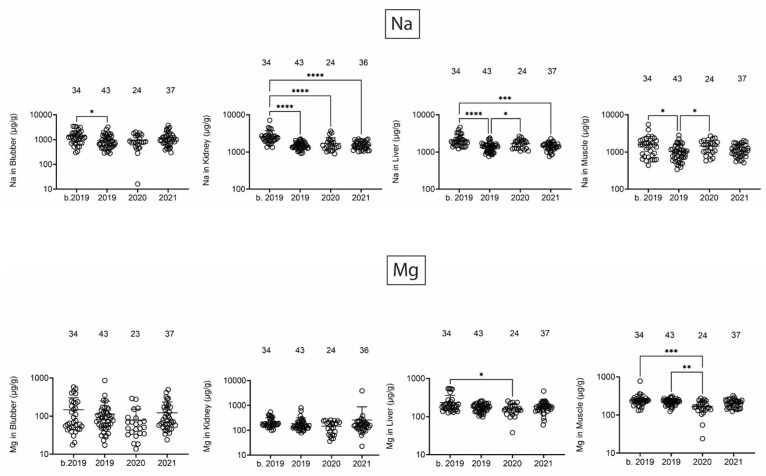
Na and Mg concentrations (µg/g) in blubber, kidney, liver, and muscle. Numbers above scatter dots represent the number of bottlenose dolphins with the metal/metalloid in their tissue. Total sample size: b. 2019 = 42; 2019 = 43; 2020 = 24; 2021 = 37. *, **, ***, **** difference between indicated groups at *p* < 0.05, 0.01, 0.001, or 0.0001, respectively.

Na and Mg did reveal some changes that might be attributed to the dual opening of the BCS and/or the UME in 2019 (Figure 1). There was a decrease in Na from b. 2019 to 2019 in blubber and a trend toward relatively lower Mg concentrations in 2019 and 2020. In kidney, Na was lower in all dolphins that stranded starting in 2019. In liver and muscle, Na was at the lowest concentration in 2019, and Mg was lowest in 2020. Interestingly, the one consistent finding across tissues was that Na tissue levels, in general, were highest in dolphins that stranded b. 2019.

### 3.2. Na and Mg Tissue Concentrations Correlate with MSS Salinity

**Figure 2:**
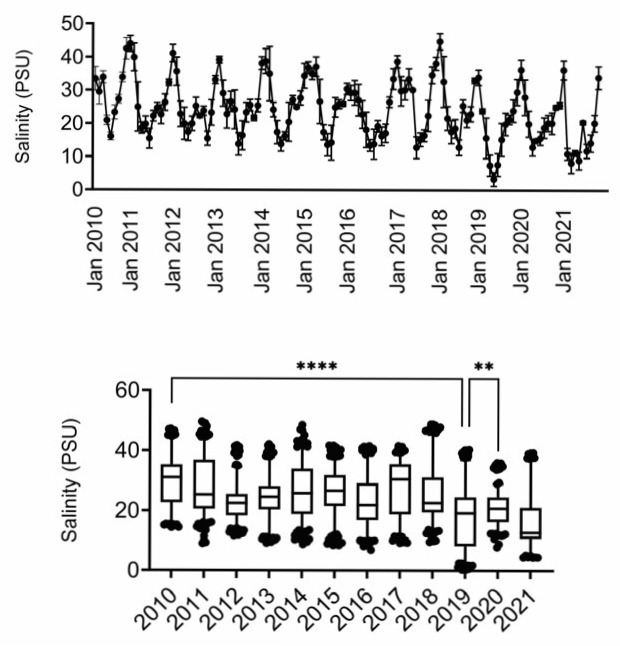
Salinity measures in MSS. Data were obtained from USGS. Timeline depicts average salinity per month, with peaks in winter months and troughs in summer months. Box and whisker plots depict mean and 95% confidence intervals. **, **** difference as compared to 2019 (only 2019 and 2021 were not different). PSU, practical salinity units.

Based on these observations, we conducted a correlation analysis for two elements that are also important components in salinity (i.e., Na and Mg) [20] to determine if Na or Mg tissue levels correlated with average yearly salinity in the MSS. We obtained average salinity data from the MSS at the US Geological Society Gulfport Light water quality monitoring station (301912088583300) [21]. As seen in Figure 2, salinity in the MSS exhibits seasonal fluctuations (lowest in summer months) and overall has been decreasing over time.

**Figure 3:**
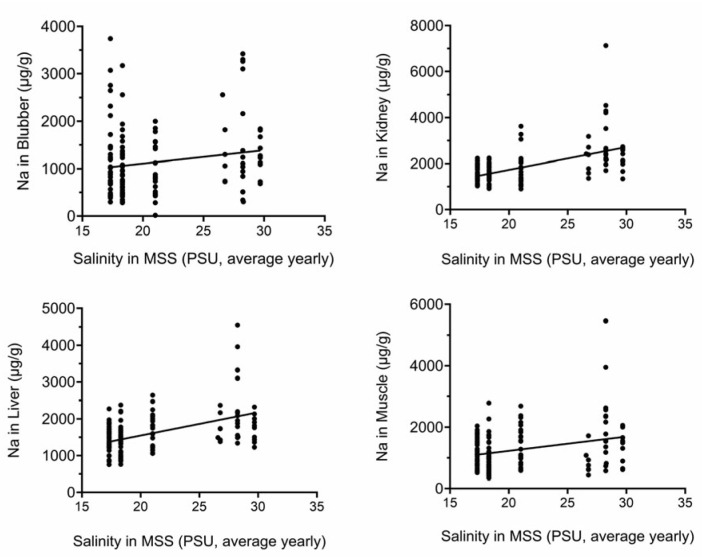
Na tissue concentrations correlate with yearly average salinity in MSS. Na concentrations (µg/g) were plotted against yearly average salinity (PSU). All correlations were significant. PSU, practical salinity units.

**Figure 4:**
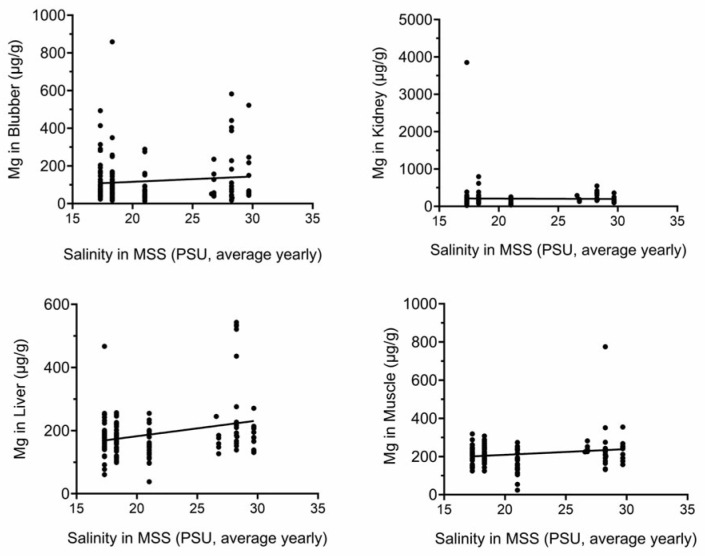
Mg tissue concentrations correlate with yearly average salinity in MSS. Mg concentrations (µg/g) were plotted against yearly average salinity (PSU). Correlations in liver and muscle were significant. PSU, practical salinity units.

Figure 3 and Figure 4 show that there are significant positive correlations between average yearly water salinity in the MSS with Na in all tissues and with Mg in liver and muscle.

### 3.3. Heavy Metal Analysis

**Figure 5:**
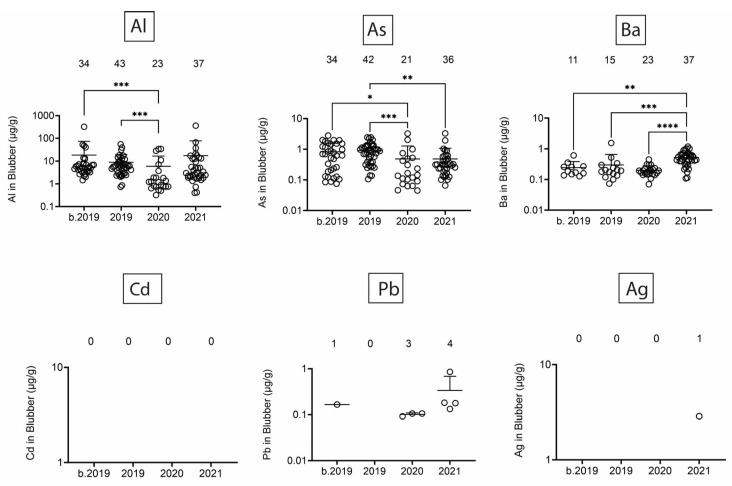
Toxic/heavy metal/metalloid (Al, As, Ba, Cd, Pb, Ag) concentrations (µg/g) in blubber. Numbers above scatter dots represent the number of bottlenose dolphins with the metal/metalloid in their tissues. Total sample size: b. 2019 = 42; 2019 = 43; 2020 = 24; 2021 = 37. *, **, ***, **** difference between indicated groups at *p* < 0.05, 0.01, 0.001, or 0.0001, respectively.

**Figure 6:**
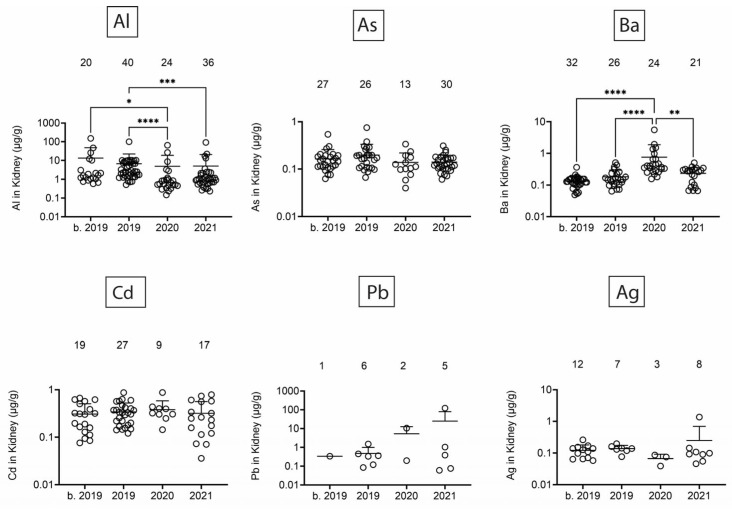
Toxic/heavy metal/metalloid (Al, As, Ba, Cd, Pb, Ag) concentrations (µg/g) in kidney. Numbers above scatter dots represent the number of bottlenose dolphins with the metal/metalloid in their tissues. Total sample size: b. 2019 = 42; 2019 = 43; 2020 = 24; 2021 = 36. *, **, ***, **** difference between indicated groups at *p* < 0.05, 0.01, 0.001, or 0.0001, respectively.

**Figure 7:**
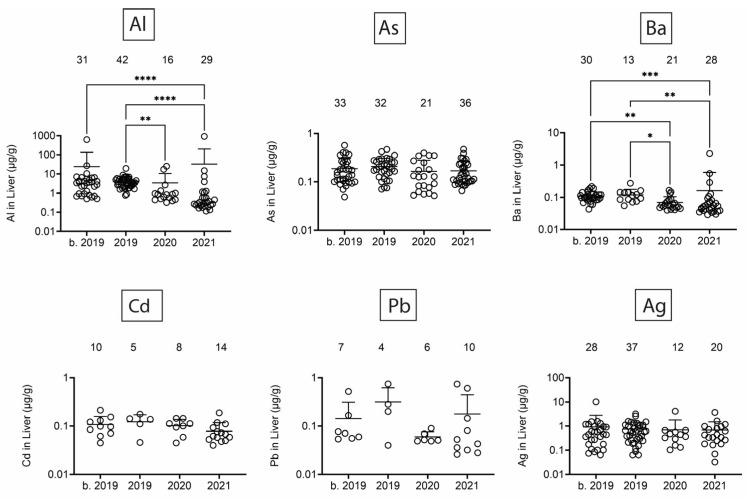
Toxic/heavy metal/metalloid (Al, As, Ba, Cd, Pb, Ag) concentrations (µg/g) in liver. Numbers above scatter dots represent the number of bottlenose dolphins with the metal/metalloid in their tissues. Total sample size: b. 2019 = 42; 2019 = 43; 2020 = 24; 2021 = 37. *, **, ***, **** difference between indicated groups at *p* < 0.05, 0.01, 0.001, or 0.0001, respectively.

**Figure 8:**
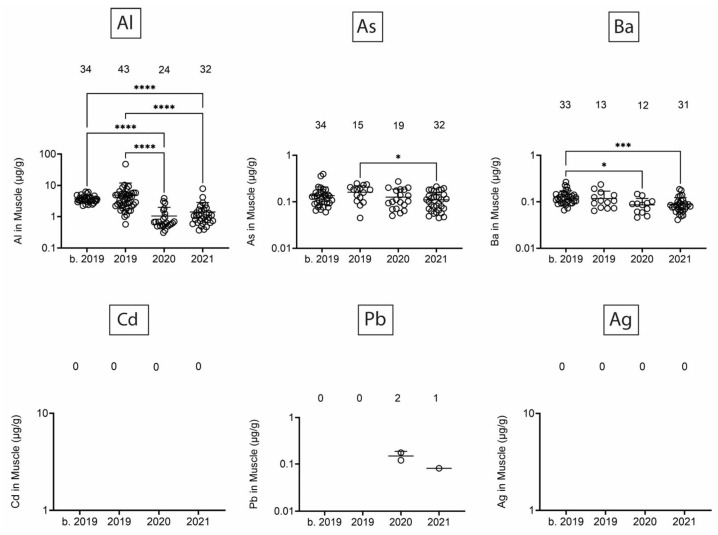
Toxic/heavy metal/metalloid (Al, As, Ba, Cd, Pb, Ag) concentrations (µg/g) in muscle. Numbers above scatter dots represent the number of bottlenose dolphins with the metal/metalloid in their tissues. Total sample size: b. 2019 = 42; 2019 = 43; 2020 = 24; 2021 = 37. *, ***, **** difference between indicated groups at *p* < 0.05, 0.01, or 0.0001, respectively.

Next, we assessed heavy/toxic metals in tissues over time. There was little Pb and Ag and no Cd in blubber (Figure 5). In blubber, Al was lowest in 2020, As was lower in 2020 and 2021 as compared to earlier times, and Ba was detected at the highest levels in dolphins that stranded in 2021. All six toxic metals were detected in kidney (Figure 6), but only Al and Ba exhibited significant T-D differences, with Al being lower in 2020 and 2021 as compared to earlier times, and Ba being highest in dolphins that stranded in 2020. All six toxic metals were also detected in liver (Figure 7), and again, T-D differences were only noted with Al and Ba. Again, Al was lower in 2020 and 2021 as compared to earlier times, and Ba was trending up in later years. Similar to blubber, there was little to no Cd, Pb, or Ag in muscle (Figure 8). Al and Ba were higher in tissues from dolphins that stranded b. 2019, and there was a decrease in As in 2021 as compared to 2019.

**Figure 9:**
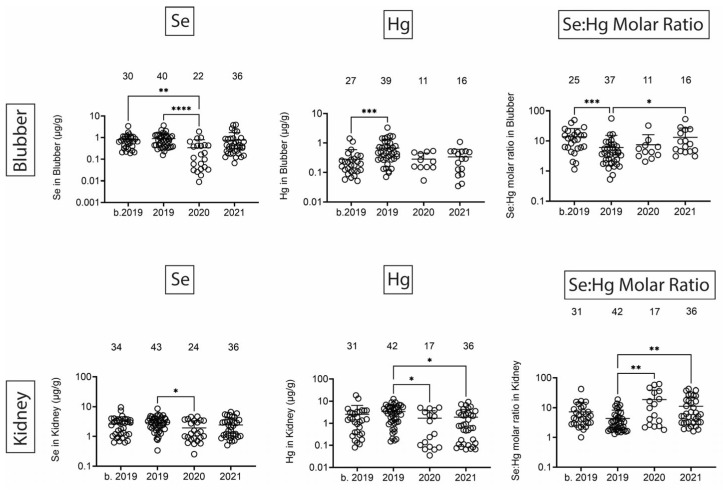
Se and Hg concentrations (µg/g) in blubber and kidney. Numbers above scatter dots represent the number of bottlenose dolphins with Se or Hg in their tissues. Plots on the right side are molar ratios of Se:Hg. Total sample size: b. 2019 = 42; 2019 = 43; 2020 = 24; 2021 = 37 for liver, 36 for kidney. *, **, ***, **** difference between indicated groups at *p* < 0.05, 0.01, 0.001, or 0.0001, respectively.

**Figure 10:**
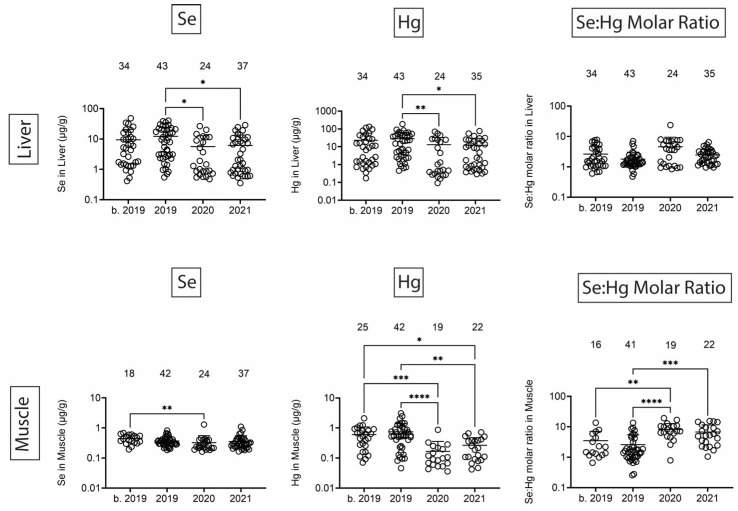
Se and Hg concentrations (µg/g) in liver and muscle. Numbers above scatter dots represent the number of bottlenose dolphins with Se or Hg in their tissues. Plots on the right side are molar ratios of Se:Hg. Total sample size: b. 2019 = 42; 2019 = 43; 2020 = 24; 2021 = 37. *, **, ***, **** difference between indicated groups at *p* < 0.05, 0.01, 0.001, or 0.0001, respectively.

**Figure 11:**
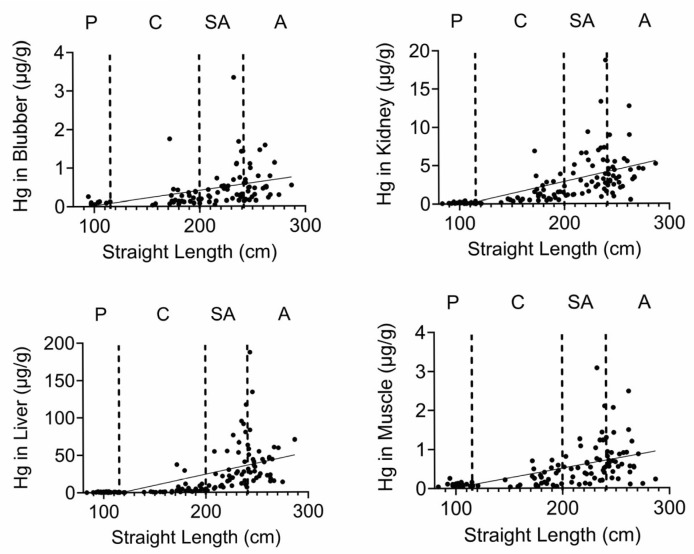
Hg tissue concentrations correlate with straight length. Hg concentrations (µg/g) were plotted against dolphin length (cm). All correlations were significant.

Finally, we quantified Se, Hg, and the Se:Hg molar ratio. Se has the potential to reduce Hg toxicity through the formation of insoluble Se:Hg complexes called tiemmanite [22], which reduces Hg availability to engage other biological targets. There were several T-D effects on Se and Hg levels in all tissues (Figure 9 and Figure 10). Focusing on the potential to form tiemmanite complexes, the Se:Hg ratio was higher in 2020 and 2021 as compared to 2019 in all tissues, suggesting the possibility of lower Hg toxicity due to higher Se:Hg ratios in the later years. As one example of toxic metal bioaccumulation, we noted that Hg concentrations were directly correlated with dolphin straight length in all tissues (Figure 11).

## 4. Discussion

This work was an extension of our initial analysis of the putative association of chemical levels of tissues in dolphins that stranded before and after the 2019 UME in the MSS. Our original work showed few changes in environmental contaminants (i.e., PCBs and PAHs) in the tissues of dolphins that stranded over the period of time surrounding the UME [15], so this work focused on time-dependent effects on metals and metalloids in blubber, kidney, liver, and muscle.

One of the most interesting findings was the relatively high levels of Na in all four tissues in the samples obtained before 2019. As previously noted, the 2019 UME was the result of an extensive freshwater incursion into Lake Pontchartrain and the MSS [14]. Many of the dolphins that stranded during the UME died in part due to sepsis associated with freshwater skin lesions and electrolyte imbalances, including hyponatremia [23]. While we did not measure Na blood levels nor could we assess organ function in our stranded dolphins, the relatively lower Na in the tissues since 2019 suggests there might be persistent, relatively low salinity in the MSS (although not so low as to cause persistent UMEs). It was interesting to note that Mg was also relatively high in dolphins that stranded before 2019, especially in liver and muscle, and that Mg is an important contributor to salinity in seawater [20] besides Na, chloride, and sulfate (although the latter two were not measured in our dolphin tissues). Ca and K also contribute to salinity [20], and while there were few tissue changes in K levels, Ca in muscle and liver were lower in 2021 as compared to before 2019. Therefore, there is a trend for lower Na, Mg, and Ca in various tissues after 2019, which might be due to salinity changes, as suggested by several regression analyses of Na and Mg. Various models from the freshwater incursion in 2019 clearly demonstrate that salinity was low in the MSS at that time [24,25,26], and water quality monitoring in the MSS by the USGS has demonstrated decreased salinity over time [21].

We also noted the presence of several toxic and heavy metals and metalloids. While Al, As, Ba, Se, and Hg were detected in all tissues, Cd, Pb, and Ag were primarily found in kidney and liver, of which the kidney is considered the main organ that accumulates Cd [27]. The Cd results are similar to Shoham-Frider et al. and Capelli et al., in which Cd was detected in these two organs [28,29]. Capelli et al. attributed the high Cd value detected in *Grampus griseus* (Risso’s dolphins) to the consumption of squids, which are generally rich in Cd [28,30]. However, the majority of the bottlenose dolphin population in the MSS has a diet consisting of soniferous prey species [31], suggesting the possibility that the accumulated Cd might also come from another source distinct from their food.

It was not surprising that As was readily detected in all tissues. It has been reported that As is widespread in marine environments, and although the liver is the main site of storage and metabolism for As [32], we did not detect differences in the average levels across tissues or time for As. As exists in various oxidation states and can be in inorganic or organic forms; it can also bioaccumulate in aquatic species [33]. In vivo, it can produce cell and tissue damage, immunotoxicity, growth inhibition, and reduction in fecundity, which might be due to its ability to induce apoptosis or oxidative stress [33].

Ba levels in blubber, kidney, and liver were higher in later years, while in muscle, Ba was decreased over time. Although the significance of the changes in Ba over time is not entirely clear, they might reflect changes from oil or gas drilling, which contributes to the release of various Ba species from the seabed [34]. In another study, Ba was detected in muscle in two other dolphin species (*Sousa plumbea* and *Tursiops aduncus*, Indian Ocean humpback dolphin and Indo-Pacific bottlenose dolphin) off the coast of South Africa [35]. The muscle Ba levels, although provided in dry weight as opposed to ours in wet weight, were similar to our values [35].

Ag accumulated in liver, as exemplified by both the average level and number of livers that were positive for Ag as compared to kidney, blubber, and muscle. In *Steno bredanensis* (rough-toothed) dolphins that had beached on the gulf side of Florida, Ag levels were also highest in liver, and there was evidence of Ag bioaccumulation in liver with age [36]. Al was also detected in all tissues, although levels of Al in tissues in marine mammals are not as broadly reported as other toxic metals, which might be due to the fact that it accumulates in bone [37] and is therefore not assessed as often as soft tissues. In our study, Al levels were lower in dolphins that stranded after 2019. Whether this decrease represents lower exposures as a result of dilution of Al concentrations following the freshwater incursion from 2019 or possible redistribution to bone from muscle is not known.

Se and Hg were also detected in all tissues. Hg levels exhibited various T-D changes in all tissues. We also observed some decreases in Se in 2020 in all tissues. Interestingly, the molar ratios of Se:Hg were increased in 2020 and 2021 as compared to 2019 in most tissues. This might suggest the dolphins were relatively protected from some of the Hg effects due to the possible formation of the tiemmanite complexes in the later years [22]. However, it should be noted that we quantified total Hg (THg), but the most toxic form is monomethyl-Hg (MeHg+), which has the potential to bioaccumulate in blood, skin, liver, kidney, and muscle [38]. Indeed, we found a significant positive correlation between Hg concentrations and dolphin straight length in all tissues. The health effects of THg on live bottlenose dolphins might result in pathophysiological impacts on various organ systems, including blood cell production, endocrine, liver, and kidney tissues [38]. Other studies have also focused on quantification of Hg levels in dolphins, although relatively fewer studies have done so in healthy free-ranging dolphins, except for health assessment projects with live capture and release studies conducted on the east and west coasts of Florida [39,40].

Since marine mammal contamination by Hg is such a cause for concern, we can compare our values to other studies. For instance, in stranded bottlenose dolphins, the average liver Hg levels were ~16, 39, and 18 mg/kg (equal to µg/g) for dolphins that stranded off the Eastern Australia, US Atlantic, or South Carolina coasts, respectively [41,42,43]. Our total average liver Hg level, regardless of time, was ~20 µg/g, so these levels were all similar to the levels in dolphins that stranded in the MSS. However, more recent studies show that average liver Hg levels were ~247 µg/g dry weight from blubber plus liver tissues of dolphins stranded in the southeastern US from 2010 to 2018 [32], ~318 µg/g dry weight from liver of adult dolphins stranded off the Virginia coast [44], and ~110 µg/g dry weight from livers from adult dolphins stranded off the Florida coast from 2016 to 2021 [45]. Given that the wet weight to dry weight ratio averages about 4–6 [46], these values are higher than our average wet weight Hg value of ~20 µg/g. Several studies also examined THg in skin [44,47] and one study suggested that THg levels in skin and liver can be used as biomarkers to estimate levels in other organs, such as the brain [44].

Sankar et al. conducted a comprehensive study investigating seasonal variations in trace elements, nutrients, dissolved organic matter (DOM), and carbonate system parameters over the largest deteriorating oyster reef in the Western MSS [48]. The study found elevated concentrations of trace elements (e.g., As, Cu, Pb, Zn, U, and Ca) during the winter of 2018 compared to summer 2019. They attributed the higher concentrations in 2018 to the increased freshwater input from greater precipitation in summer 2019 [48]. Our results for these same metals/metalloids only showed that Ca and Cu were lowest in 2019 in liver, suggesting minor effects of water trace elements on tissue levels from dolphins that stranded in the same timeframe.

## 5. Conclusions

Overall, these data show that bottlenose dolphins that strand in the MSS have detectable amounts of toxic and heavy metals and metalloids in their tissues. There were only a few toxic metals/metalloids with lower tissue levels after the UME in 2019. However, there was a consistent trend that the Se:Hg ratio increased after 2019, suggesting possibly higher Se exposure, lower Hg exposure, or increased potential for Se:Hg complex formation after the UME. Interestingly, Na and Mg levels were lower in several tissues after the 2019 UME, and there were significant correlations between Na and Mg tissue levels with average yearly salinity data. It is important to emphasize that we cannot attribute the strandings or dolphin mortalities to the presence or levels of these metals and metalloids, but these data demonstrate that we should continue to monitor these apex predators as indicators of marine, and possibly, human exposures to these metals and metalloids.

## Figures and Tables

**Table 1:** Descriptive effects of elements in the tissues of dolphins that stranded in the MSS from 2010 to 2021.

Metal/ Metalloid	Tissue	Time-Dependent (T-D) Effect	Description of T-D Effects
Ca	Blubber		No T-D effects in blubber or kidney; liver lowest concentration in 2019; muscle lowest concentration in 2021
	Kidney		
	Liver	•	
	Muscle	•	
Fe	Blubber	•	No T-D effects in kidney or liver; blubber and muscle lowest concentration in 2020
	Kidney		
	Liver		
	Muscle	•	
P	Blubber		No T-D effects in blubber, kidney, or liver; muscle slight elevation from 2019 to 2020
	Kidney		
	Liver		
	Muscle	•	
K	Blubber		No T-D effects in blubber, liver, or muscle; kidney slight decrease in 2019 as compared to b. 2019
	Kidney	•	
	Liver		
	Muscle		
Zn	Blubber	•	No T-D effects in liver or muscle; blubber lower concentrations in time periods before 2019 as compared to 2020 and 2021; kidney highest concentration in 2021
	Kidney	•	
	Liver		
	Muscle		
Cr	Blubber	•	No T-D effects in muscle; blubber lowest concentration in 2020 and relatively fewer dolphins positive in 2020 and 2021; kidney highest concentration in 2019; liver highest concentration b. 2019
	Kidney	•	
	Liver	•	
	Muscle		
Cu	Blubber	•	No T-D effects in kidney or muscle; blubber higher concentrations in 2020 and 2021; liver lowest concentration in 2019
	Kidney		
	Liver	•	
	Muscle		
Ni	Blubber		No T-D effects in blubber, kidney, or liver; muscle highest concentration in 2019 and relatively fewer dolphins positive in 2020 and 2021
	Kidney		
	Liver		
	Muscle	•	
Mn	Blubber		No T-D effects in blubber or kidney; liver concentration lowest in 2020; muscle concentration highest in 2019
	Kidney		
	Liver	•	
	Muscle	•	

• indicates a statistically significant time-dependent (T-D) effect detected in at least one timeframe, as shown in Supplemental Figures S1–S8.

## Data Availability

All data are provided either in the manuscript or as supplemental files.

## Supplementary Materials

The following supporting information can be downloaded at: https://www.mdpi.com/article/10.3390/toxics13060511/s1, Figure S1: Metal/metalloid (Ca, Fe, P, K, Mn, and Zn) concentrations (µg/g) in blubber; Figure S2: Metal/metalloid (Cr, Cu, and Ni) concentrations (µg/g) in blubber; Figure S3: Metal/metalloid (Ca, Fe, P, K, Mn, and Zn) concentrations (µg/g) in kidney; Figure S4: Metal/metalloid (Cr, Cu, and Ni) concentrations (µg/g) in kidney; Figure S5: Metal/metalloid (Ca, Fe, P, K, Mn, and Zn) concentrations (µg/g) in liver; Figure S6: Metal/metalloid (Cr, Cu, and Ni) concentrations (µg/g) in liver; Figure S7: Metal/metalloid (Ca, Fe, P, K, Mn, and Zn) concentrations (µg/g) in muscle; Figure S8: Metal/metalloid (Cr, Cu, and Ni) concentrations (µg/g) in muscle; Table S1: Raw values for all graphs.

## Abbreviations

The following abbreviations are used in this manuscript:

BCS	Bonnet Carré Spillway
MSS	Mississippi Sound
UME	Unusual mortality event
T-D	Time-dependent

## References

[1] Jomova K., Makova M., Alomar S.Y., Alwasel S.H., Nepovimova E., Kuca K., Rhodes C.J., Valko M. (2022). Essential metals in health and disease. Chem. Biol. Interact..

[2] Ufelle A.C., Barchowsky A., Klaassen C. (2019). Toxic Effects of Metals. Casarett and Doull’s Toxicology.

[3] Vincent J.B. (2017). New Evidence against Chromium as an Essential Trace Element. J. Nutr..

[4] Tchounwou P.B., Yedjou C.G., Patlolla A.K., Sutton D.J. (2012). Heavy metal toxicity and the environment. Exp. Suppl..

[5] Robson A.J., Neal C. (1997). A summary of regional water quality for Eastern UK rivers. Sci. Total Environ..

[6] Shahidul Islam M., Tanaka M. (2004). Impacts of pollution on coastal and marine ecosystems including coastal and marine fisheries and approach for management: A review and synthesis. Mar. Pollut. Bull..

[7] Davies A.G. (1979). Pollution studies with marine plankton: Part II. Heavy metals. Adv. Marine Biol..

[8] Saidon N.B., Szabo R., Budai P., Lehel J. (2024). Trophic transfer and biomagnification potential of environmental contaminants (heavy metals) in aquatic ecosystems. Environ. Pollut..

[9] Garrison L.P., Ortega-Ortiz J., Rappucci G. (2021). Abundance of Coastal and Continental Shelf Stocks of Common Bottlenose and Atlantic Spotted Dolphins in the Northern Gulf of Mexico: 2017-2018. https://repository.library.noaa.gov/view/noaa/43721.

[10] Miller L., Mackey A., Solangi M., Kuczaj S. (2013). Population abundance and habitat utilization of bottlenose dolphins in the Mississippi Sound. Aquat. Conserv. Mar. Freshw. Ecosyst..

[11] Pitchford J.L., Pulis E.E., Evans K., Shelley J.K., Serafin B.J.S., Solangi M. (2016). Seasonal Density Estimates of *Tursiops truncatus* (Bottlenose Dolphin) in the Mississippi Sound from 2011 to 2013. Southeast. Nat..

[12] US Army Corps of Engineers (2014). Bonnet Carre Spillway. https://www.mvn.usace.army.mil/Portals/56/docs/PAO/Brochures/BCspillwaybooklet.pdf.

[13] US Army Corps of Engineers Spillway Operation Information. https://www.mvn.usace.army.mil/Missions/Mississippi-River-Flood-Control/Bonnet-Carre-Spillway-Overview/Spillway-Operation-Information.

[14] NOAA 2019 Bottlenose Dolphin Unusual Mortality Event Along the Northern Gulf of Mexico. https://www.fisheries.noaa.gov/national/marine-life-distress/2019-bottlenose-dolphin-unusual-mortality-event-along-northern-gulf.

[15] Landrau-Giovannetti N., Rogers J., Murray R., Reichley S., Moore D., Madrigal T., Brown A., Meredith A., Childers C., Sparks D. (2024). Determination of PCB and PAH Tissue Levels in Bottlenose Dolphins that Stranded in the Mississippi Sound Before and After the Unusual Mortality Event in 2019. Sci. Total Environ..

[16] NOAA (2021). Level A Data Collection for Marine Mammal Stranding Events. https://www.fisheries.noaa.gov/national/marine-life-distress/level-data-collection-marine-mammal-stranding-events.

[17] Graur D., Higgins D.G. (1994). Molecular Evidence for the Inclusion of Cetaceans within the Order Artiodactyla. Mol. Biol. Evol..

[18] Thewissen J.G.M., Cooper L.N., George J.C., Bajpai S. (2009). From Land to Water: The Origin of Whales, Dolphins, and Porpoises. Evol. Edu. Outreach.

[19] Tomza-Marciniak A., Pilarczyk B., Drozd R., Pilarczyk R., Juszczak-Czasnojc M., Havryliak V., Podlasinska J., Udala J. (2023). Selenium and mercury concentrations, Se:Hg molar ratios and their effect on the antioxidant system in wild mammals. Environ. Pollut..

[20] Riser S., NASA: Salinity Sea Surface Salinity from Space. https://salinity.oceansciences.org/science-salinity.htm#:~:text=In%20the%20table%20are%20the,we’re%20talking%20about%20everything.

[21] USGS (2021). National Water Information System Data Available on the World Wide Web (USGS Water Data for the Nation). https://waterdata.usgs.gov/monitoring-location/301912088583300/#parameterCode=00065&period=P7D&showMedian=false.

[22] Palmisano F., Cardellicchio N., Zambonin P.G. (1995). Speciation of mercury in dolphin liver: A two-stage mechanism for the demethylation accumulation process and role of selenium. Marine Environ. Res..

[23] Hughes S.M., McNulty K.C., Morgan T.W. Dolphin Lesions Associated with Freshwater Incursions. Proceedings of the International Association for Aquatic Animal Medicine.

[24] Armstrong B.N., Cambazoglu M.K., Wiggert J.D. Modeling the impact of the 2019 Bonnet Carré Spillway opening and local river flooding on the Mississippi Sound. Proceedings of the OCEANS 2021: San Diego–Porto.

[25] Shahidzadehasadi M., Linhoss A., Moore D., Reichley S., Mickle P.F., Lawrence M. (2024). Examining the effect of salinity on dolphin mortality using Lagrangian particle tracking in a hydrodynamic model. Esuarine Coast. Shelf Sci..

[26] Saran S.H., Cambazoglu M.K., Armstrong B.N., Bernstein D.N., Wiggert J.D. Assessment of the Impact of the Variability of Freshwater Input and Land-Sea Breeze on the Mississippi Sound Using a Modeling Approach. Proceedings of the OCEANS 2022.

[27] Thompson D.R., Furness R.W., Rainbow P.S. (1990). Metal levels in marine vertebrates. Heavy Metals in the Marine Environments.

[28] Capelli R., Das K., Pellegrini R.D., Drava G., Lepoint G., Miglio C., Minganti V., Poggi R. (2008). Distribution of trace elements in organs of six species of cetaceans from the Ligurian Sea (Mediterranean), and the relationship with stable carbon and nitrogen ratios. Sci. Total Environ..

[29] Shoham-Frider E., Kress N., Wynne D., Scheinin A., Roditi-Elsar M., Kerem D. (2009). Persistent organochlorine pollutants and heavy metals in tissues of common bottlenose dolphin (*Tursiops truncatus*) from the Levantine Basin of the Eastern Mediterranean. Chemosphere.

[30] Storelli M.M., Zizzo N., Marcotrigiano G.O. (1999). Heavy metals and methylmercury in tissues of Risso’s dolphin (*Grampus griseus*) and Cuvier’s beaked whale (*Ziphius cavirostris*) stranded in italy (South Adriatic sea). Bull. Environ. Contam. Toxicol..

[31] Smith C., Hurley B., Toms C., Mackey A., Solangi M., Kuczaj II S. (2013). Hurricane impacts on the foraging patterns of bottlenose dolphins Tursiops truncatus in Mississippi Sound. Mar. Ecol. Prog. Ser..

[32] Page-Karjian A., Lo C.F., Ritchie B., Harms C.A., Rotstein D.S., Han S., Hassan S.M., Lehner A.F., Buchweitz J.P., Thayer V.G. (2020). Anthropogenic Contaminants and Histopathological Findings in Stranded Cetaceans in the Southeastern United States, 2012–2018. Front. Mar. Sci..

[33] Byeon E., Kang H.M., Yoon C., Lee J.S. (2021). Toxicity mechanisms of arsenic compounds in aquatic organisms. Aquat. Toxicol..

[34] ATSDR Barium: Division of Toxicology and Human Health Services ToxFAQs. https://www.atsdr.cdc.gov/toxfaqs/tfacts24.pdf.

[35] Plon S., Roussouw N., Uren R., Naidoo K., Siebert U., Cliff G., Bouwman H. (2023). Elements in muscle tissue of three dolphin species from the east coast of South Africa. Mar. Pollut. Bull..

[36] Mackey E.A., Oflaz R.D., Epstein M.S., Buehler B., Porter B.J., Rowles T., Wise S.A., Becker P.R. (2003). Elemental composition of liver and kidney tissues of rough-toothed dolphins (*Steno bredanensis*). Arch. Environ. Contam. Toxicol..

[37] Borrell A., Garcia-Garin O., Aguilar A., Vighi M., Valdivia M., Gonzalez E.M., Paez-Rosas D., Drago M. (2023). High aluminum content in bone of marine mammals and its relation with source levels and origin. Environ. Pollut..

[38] Reif J.S., Schaefer A.M., Bossart G.D. (2015). Atlantic Bottlenose Dolphins (*Tursiops truncatus*) as A Sentinel for Exposure to Mercury in Humans: Closing the Loop. Vet. Sci..

[39] Stavros H.C., Bossart G.D., Hulsey T.C., Fair P.A. (2007). Trace element concentrations in skin of free-ranging bottlenose dolphins (*Tursiops truncatus*) from the southeast Atlantic coast. Sci. Total Environ..

[40] Stavros H.C., Bossart G.D., Hulsey T.C., Fair P.A. (2008). Trace element concentrations in blood of free-ranging bottlenose dolphins (*Tursiops truncatus*): Influence of age, sex and location. Mar. Pollut. Bull..

[41] Law R.J., Morris R.J., Allchin C.R., Jones B.R., Nicholson M.D. (2003). Metals and organochlorines in small cetaceans stranded on the east coast of Australia. Mar. Pollut. Bull..

[42] Keuhl D.W., Haebler R., Potter C. (1994). Colpanar PCB and metal residues in dolphins from the U.S. Atlantic coast including Atlantic bottlenose obtained during the 1987/88 mass mortality. Chemosphere.

[43] Beck K.M., Fair P., McFee W., Wolf D. (1997). Heavy metals in livers of bottlenose dolphins stranded along the South Carolina coast. Mar. Pollut. Bull..

[44] Malcolm E.G., Coleman S.E., Smith E.M., Cooke M.E., Rice Jeff H., Ellick R.M., Volker K.M. (2023). The potential use of skin and liver as biomarkers to estimate mercury in the brain, kidney, and muscle of bottlenose dolphins (*Tursiops truncatus*). Mar. Pollut. Bull..

[45] Bielmyer-Fraser G.K., Courville J.M., Ward A., Hardie M.M. (2024). Mercury and Selenium Accumulation in the Tissues of Stranded Bottlenose Dolphins (*Tursiops truncatus*) in Northeast Florida, 2013–2021. Animals.

[46] Pierre C., Morgane T.T., Manuel R., Charles-Andre T., Carolina G., Sebastien L., Bruno E. (2017). Underestimation of chemical contamination in marine fish muscle tissue can be reduced by considering variable wet:dry weight ratios. Mar. Pollut. Bull..

[47] McCormack M.A., Nowlin W.H., Dutton J. (2022). Effect of trophic position on mercury concentrations in bottlenose dolphins (*Tursiops truncatus*) from the northern Gulf of Mexico. Environ. Res..

[48] Sankar M.S., Dash P., Lu Y., Hu X., Mercer A.E., Wickramarathna S., Beshah W.T., Sanders S.L., Arslan Z., Dyer J.L. (2023). Seasonal changes of trace elements, nutrients, dissolved organic matter, and coastal acidification over the largest oyster reef in the Western Mississippi Sound, USA. Environ. Monit. Assess..

