# Supplementary figures and images for: Tissue Element Levels and Heavy Metal Burdens in Bottlenose Dolphins That Stranded in the Mississippi Sound Surrounding the 2019 Unusual Mortality Event
Source: Toxics. 2025 Jun 18;13(6):511. doi: 10.3390/toxics13060511 (PMC12197444; doi:10.3390/toxics13060511)

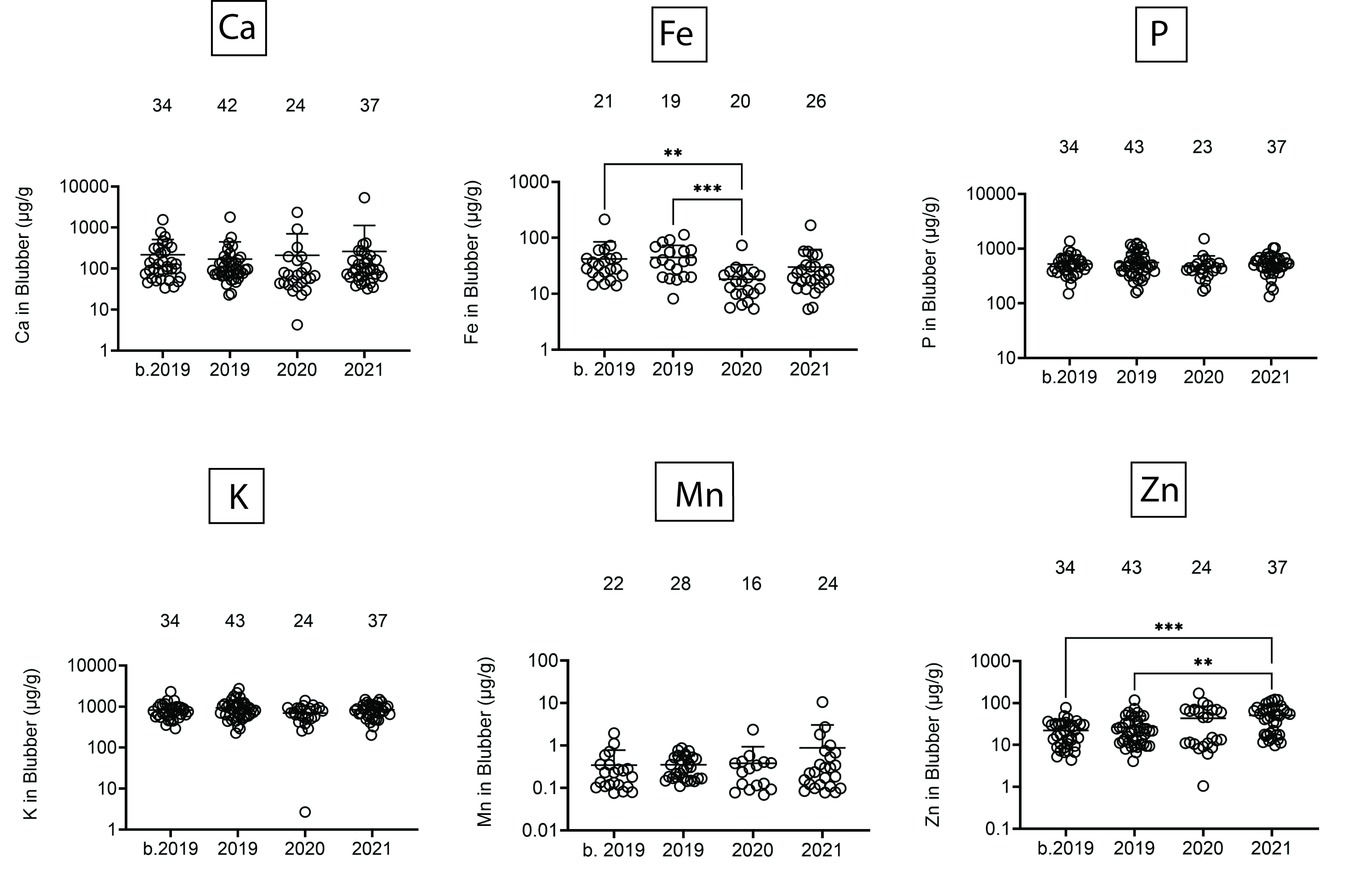

Supplement: Supplementary file 1 [file toxics-13-00511-s001.zip › resubmit with galleys/Supplemental Figure S1.tif]

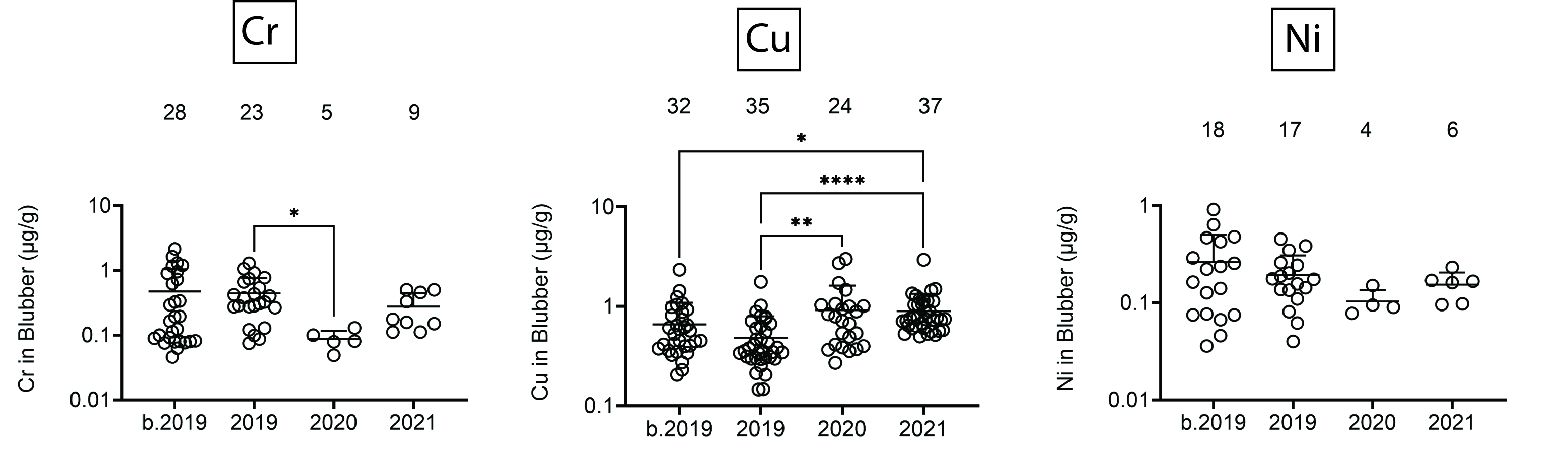

Supplement: Supplementary file 1 [file toxics-13-00511-s001.zip › resubmit with galleys/Supplemental Figure S2.tif]

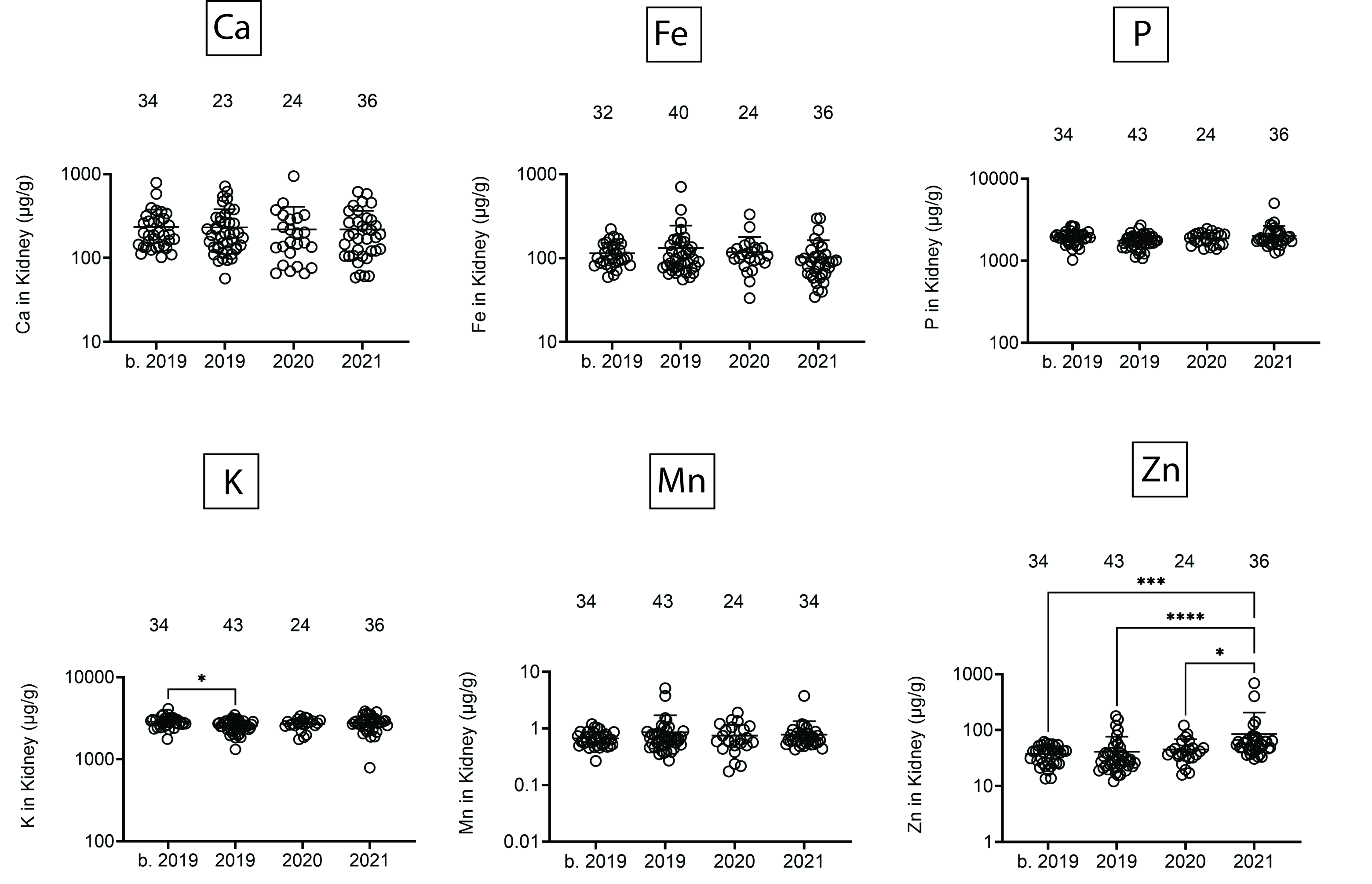

Supplement: Supplementary file 1 [file toxics-13-00511-s001.zip › resubmit with galleys/Supplemental Figure S3.tif]

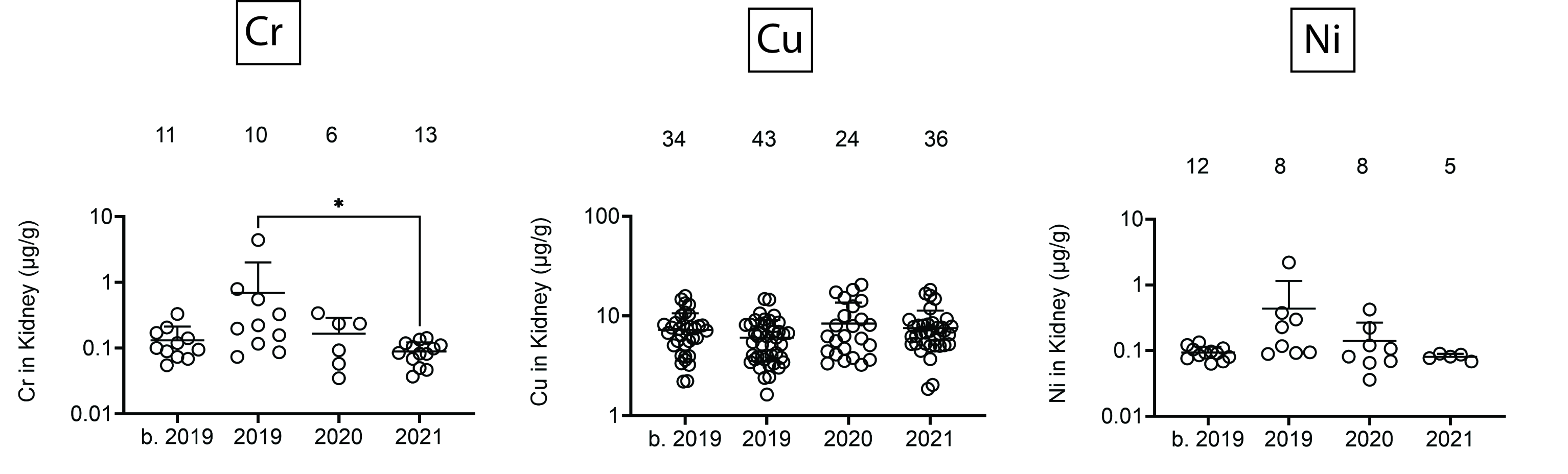

Supplement: Supplementary file 1 [file toxics-13-00511-s001.zip › resubmit with galleys/Supplemental Figure S4.tif]

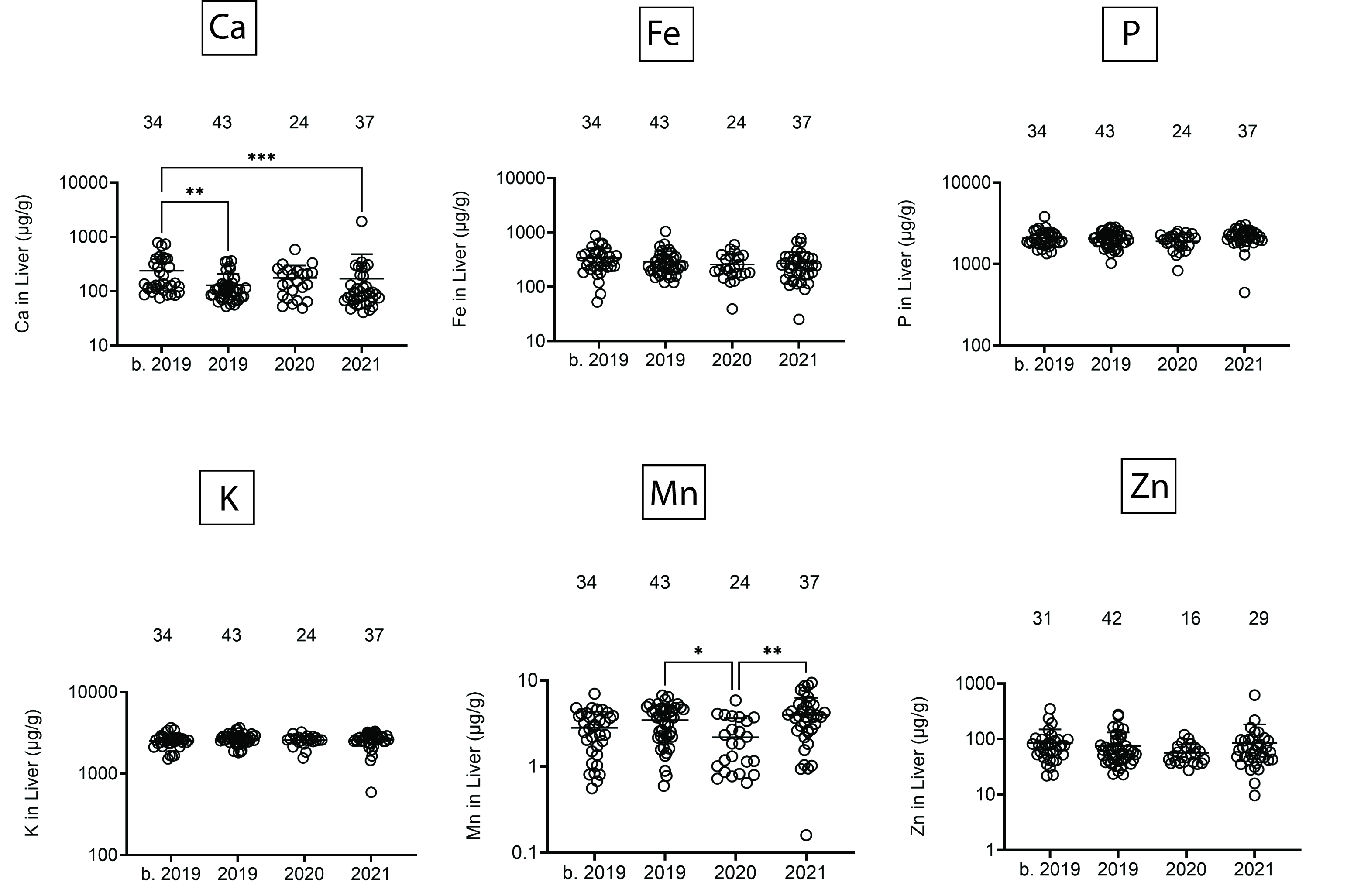

Supplement: Supplementary file 1 [file toxics-13-00511-s001.zip › resubmit with galleys/Supplemental Figure S5.tif]

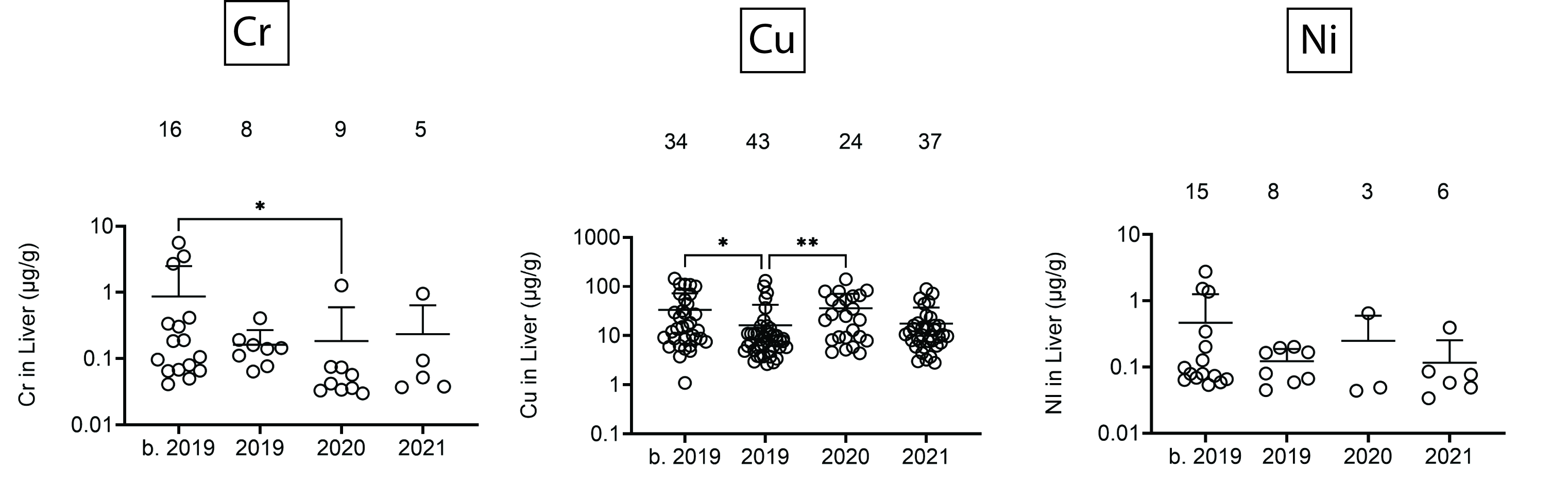

Supplement: Supplementary file 1 [file toxics-13-00511-s001.zip › resubmit with galleys/Supplemental Figure S6.tif]

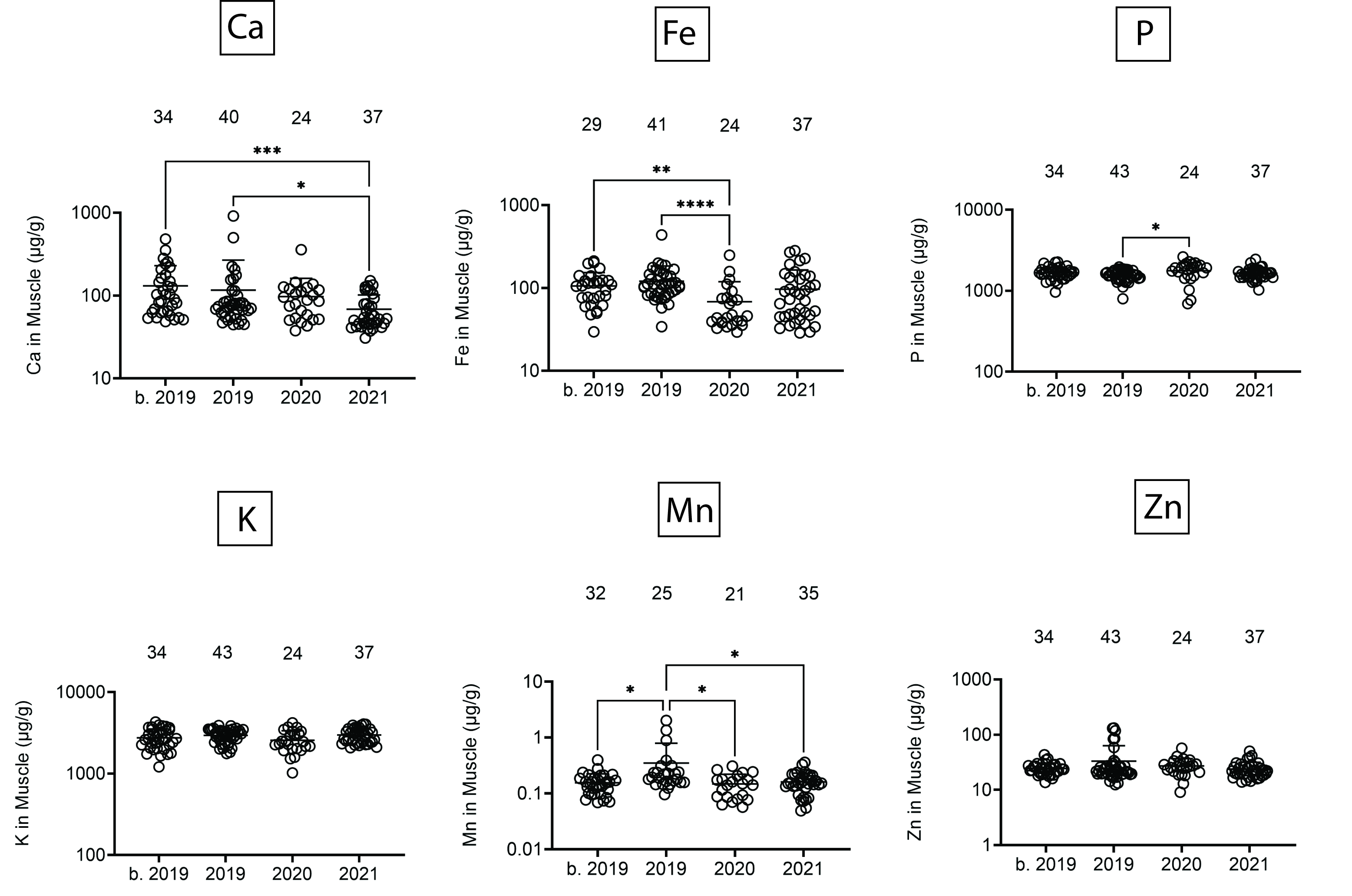

Supplement: Supplementary file 1 [file toxics-13-00511-s001.zip › resubmit with galleys/Supplemental Figure S7.tif]

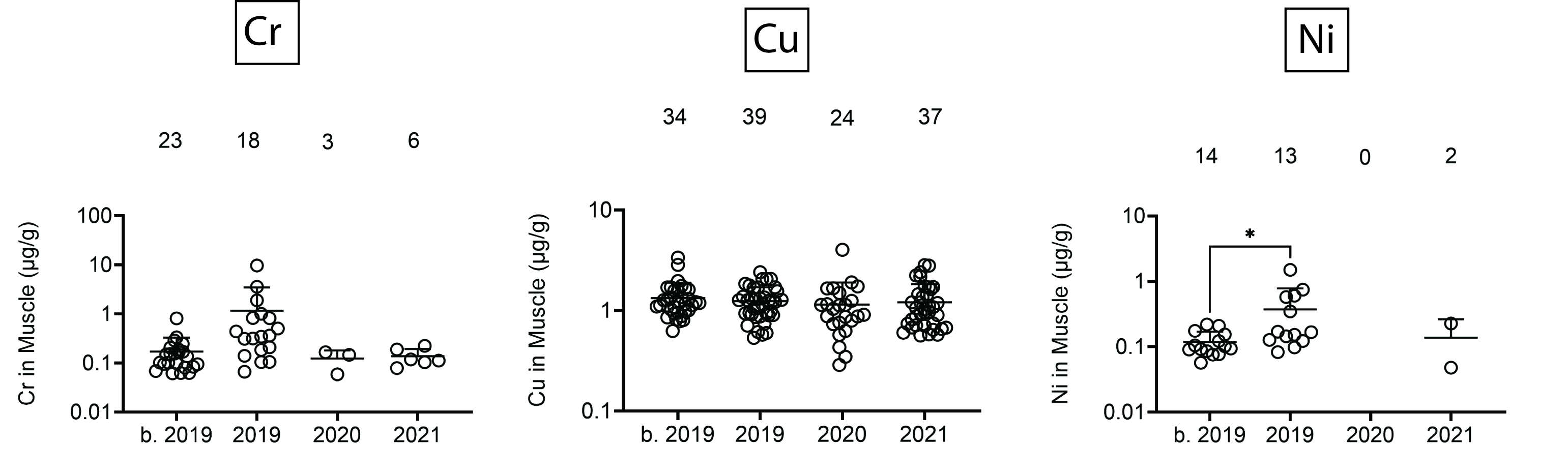

Supplement: Supplementary file 1 [file toxics-13-00511-s001.zip › resubmit with galleys/Supplemental Figure S8.tif]
